# Trends and predictions of lung cancer incidence in Jiangsu Province, China, 2009–2030: a bayesian age-period-cohort modelling study

**DOI:** 10.1186/s12885-022-10187-1

**Published:** 2022-10-31

**Authors:** Yuchen Jiang, Renqiang Han, Jian Su, Xikang Fan, Hao Yu, Ran Tao, Jinyi Zhou

**Affiliations:** 1 Department of Epidemiology, School of Public Health, Nanjing Medical University, 211166 Nanjing, China; 2 Department of Non-communicable Chronic Disease and Prevention, Jiangsu Provincial Center for Disease Control and Prevention, 210009 Nanjing, China

**Keywords:** Lung cancer, Incidence, Prediction, Modelling study

## Abstract

**Background:**

Lung cancer is currently the most frequent cancer in Jiangsu Province, China, and the features of cancer distribution have changed continuously in the last decade. The aim of this study was to analyse the trend of the incidence of lung cancer in Jiangsu from 2009 to 2018 and predict the incidence from 2019 to 2030.

**Methods:**

Data on lung cancer incidence in Jiangsu from 2009 to 2018 were retrieved from the Jiangsu Cancer Registry. The average annual percentage change (AAPC) was used to quantify the trend of the lung cancer age-standardized rate (ASR) using Joinpoint software. Bayesian age-period-cohort models were used to predict lung cancer incidence up to 2030.

**Results:**

In Jiangsu, the lung cancer crude rate increased from 45.73 per 100,000 in 2009 to 69.93 per 100,000 in 2018. The lung cancer ASR increased from 29.03 per 100,000 to 34.22 per 100,000 during the same period (AAPC = 2.17%, 95% confidence interval [CI], 1.54%, 2.80%). Between 2019 and 2030, the lung cancer ASR is predicted to decrease slightly to 32.14 per 100,000 (95% highest density interval [HDI], 24.99, 40.22). Meanwhile, the ASR showed a downward trend in males and rural regions while remaining stable in females and urban regions.

**Conclusion:**

We predict that the incidence of lung cancer in Jiangsu will decrease in the next 12 years, mainly due to the decrease in males and rural areas. Therefore, future lung cancer prevention and control efforts should be focused on females and urban regions.

## Introduction

Lung cancer is the leading type of cancer with the highest incidence and mortality in China and the world. It is estimated that more than 820,000 new lung cancer cases occurred in China in 2020 [1]. According to data from the Jiangsu Cancer Registry, in 2016, lung cancer was the malignant tumour with the highest incidence rate in Jiangsu Province, with ASR of 33.37/100,000, which was lower than the national average (36.46/100,000) [2, 3]. However, there is a lack of analysis and prediction on lung cancer in Jiangsu Province by sex and region. In a previous the Global Burden of Disease (GBD) study, tobacco smoking and industrially emitted ambient matter pollution were reported to be the leading risk factors for lung cancer [4]. Male smoking rates remain high in Jiangsu today; in contrast, the tobacco epidemic is less evolved and defined among females [5]. Derived from rapid growth and industrialization, air pollution is more severe in cities than in rural areas [6]. Therefore, it is important to analyse and predict lung cancer trends by sex and region.

Through mathematical models, past surveillance data can be used to predict the future cancer disease burden based on the assumption that recent incidence trends will continue to some extent in the future. In particular, Bayesian age-period-cohort models (BAPC models) have proven to be an effective tool for analysing and predicting trends of incidence [7–9]. The age effect accounts for the duration of exposure to risk variables. The period effect refers to circumstances that affect all persons at the same time, irrespective of age (e.g., short-term exposure, behaviour modification, therapeutic improvement). The cohort impact relates to a generational exposure [10, 11]. BAPC models are generally based on Poisson regression models, with the age effect considered as a row variable, the period effect as a column variable, and the cohort effect as the cross-effect of the age effect and the period effect, to construct a three-factor model of age, period and cohort and perform analysis and Markov chain Monte Carlo (MCMC) simulation [12, 13]. Given that, we intended to use BAPC modelling for the long-term prediction of incidence in this study.

Our study analysed and predicted the incidence of lung cancer through 2030 based on lung cancer data from Jiangsu Province from 2009 to 2018. The objectives of this study were (a) to provide an estimate of the burden of lung cancer in Jiangsu, China, using cancer registry data and (b) to provide detailed age-specific lung cancer incidence estimates through 2030.

## Materials and methods

### Source of the data

Cancer incidence data from 2009 to 2018 were obtained from the Jiangsu Cancer Registry, which is one of the largest population-based cancer registries in China. Incident cases of cancer were coded according to the International Classification of Diseases for Oncology, 3rd edition (ICD-O-3) [14] and the International Statistical Classification of Diseases 10th Revision (ICD-10) [15]. Lung cancer was defined as ICD-10 codes C34.0-C34.9. The detailed variables of each case were collected, including year and patient age at diagnosis, sex and region (rural or urban area). Age was divided into 18 subgroups, starting with 0–4 years, then 5–9 years and then in 5-year age groups up to 80–84 years, and finally 85 years or older. Based on the data-quality criteria of “Guideline for Chinese Cancer Registration” and International Agency for Research on Cancer/International Association of Cancer Registries (IARC/IACR), data quality of every cancer registry was assessed, including the validity, reliability, completeness and comparability [16]. The quality control indexes include the mortality to incidence ratio (M/I), the proportion of morphological verification (MV%), the percentage of cases identified with death certification only (DCO%) and the stability of cancer incidence. Qualified data from 16 continuous population-based cancer registries, providing a population coverage of about 17.36 million people, approximately 22.19% of the Jiangsu Province population were accepted for analysis. Seven of these registries are included in Cancer Incidence in Five Continents (CI5) Vol XI.

### Statistical analysis

#### Quantifying the incidence trend of lung cancer

To synthetically evaluate average trends that include multiple intervals, the average annual percentage change (AAPC) was used to quantify the temporal trends of the age-standardized lung cancer incidence rate (ASR) in 2009–2018 and 2019–2030, which indicate the past trends and future trends, respectively. The ASR was calculated according to the direct method using the World Standard Population as proposed by Segi [17]. The AAPC was estimated by Joinpoint 4.9.0.1 [18]. A maximum number of 2 joinpoints were allowed for the ASR. A regression line was fitted to the natural logarithm of the rates, i.e., *y = α + βx + ε*, where y = ln (ASR) and x = calendar year, and the AAPC was calculated as 100 × *(exp(β)-1)* [19].

## Bayesian age-period-cohort model

The temporal trends in age-adjusted incidence rates were modelled using the BAPC method. In the following, let *i* = 1…, *I* denote the index of the age group, *j* = 1…, *J* denote the index of the period, *k* = 1…, and *K* denote the index of the birth cohort. Given that age and period are measured on different scales (incidence data are given per year, but the age group covers 5 years), the cohort index is k = k (i, j) = 5*(I-i) + j. In classical APC literature, the APC model is often regarded as a log-linear Poisson model [20]. As an alternative, a binomial logit model can be formulated (both models are approximately identical): The counts of incidences *y*_*ij*_ in age group *i* in period *j* follow a binomial distribution with parameters *p*_*ij*_ and *n*_*ij*_. Here, *n*_*ij*_ is the known population size of age group *i* at period *j*, and *p*_*ij*_ is the unknown incidence probability. The logit of the incidence probability is decomposed into an intercept µ, age effect *θ*_*i*_, period effect *φ*_*j*_ and cohort effect *ψ*_*k*_ [20–22].

Random walk (RW) priors of different orders are used for the APC parameters age groups, periods and cohorts effect. The RW-1 prior assumes a constant trend over the time scale, whereas the RW-2 prior assumes a linear time trend [23, 24]. The results of the iterations were used to estimate the parameter values for age, period, and cohort effects based on different RW choices through Markov chain Monte Carlo (MCMC) method iterations.

This model was implemented using the Bayesian Age-Period-Cohort Modelling and Prediction package (BAMP v.1.3.0, Institute of Biomedical Engineering, Imperial College, London, UK) [25] of R version 4.0.3 (https://www.r-project.org/). Markov chain Monte Carlo simulations were run for 1,010,000 iterations with the initial 10,000 iterations used as burn-in to minimize the effect of initial values. The median iterative values and 95% confidence intervals (using 2.5% and 97.5% of the 1,000,000 iterated results, respectively) were obtained by the MCMC simulations in the models. The posterior deviance and predictive deviances of the model were used as a measure of the goodness of fit.

## Result

### Lung cancer incidence in Jiangsu, 2009–2018

In Jiangsu, the lung cancer crude rate increased from 45.73 per 100,000 to 69.93 per 100,000, and the lung cancer ASR increased from 29.03 per 100,000 to 34.22 per 100,000 during the same period (AAPC = 2.17%, 95% CI, 1.54%, 2.80%) (Table 1). The ASR increased significantly among males (AAPC = 1.30%, 95% CI, 0.83%, 1.78%) and females (AAPC = 3.94%, 95% CI, 3.09%, 4.80%). In addition, the ASR increased significantly in urban (AAPC = 1.99%, 95% CI, 1.25%, 2.74%) and rural regions (AAPC = 2.30%, 95% CI, 1.68%, 2.92%).

**Table 1:** Incidence of lung cancer from 2009 to 2018 in Jiangsu

	2009		2018		2009–2018
	Crude Rate (per 100,000)	ASR [per 100,000 (95%CI)]	Crude Rate (per 100,000)	ASR [per 100,000 (95%CI)]	AAPC of ASR [%(95% CI)]
Overall	45.73	29.03 (28.35, 29.74)	69.36	34.22 (33.59, 34.88)	2.17 (1.54, 2.80)^*^
Sex					
Male	62.33	41.46 (40.30, 42.66)	90.35	46.27 (45.23, 47.35)	1.30 (0.83, 1.78)^*^
Female	28.61	17.43 (16.69, 18.23)	47.78	23.04 (22.30, 23.83)	3.94 (3.09, 4.80)^*^
Region					
Urban	45.37	28.74 (27.58, 29.98)	66.03	33.02 (32.02, 34.09)	1.99 (1.25, 2.74)^*^
Rural	45.92	29.20 (28.36, 30.08)	71.46	34.93 (34.12, 35.79)	2.30 (1.68, 2.92)^*^

ASR: age-standardized rate, AAPC: average annual percentage change, CI: confidence interval. *, *P* < 0.001

## Age-period-cohort analyses of the incidence in Jiangsu from 2009 to 2018

BAPC models were fitted to the overall age-specific incidence rates from 2009 to 2018. Table 2 shows the change in deviance in the sequential building of the models. The full three-factor model (age-period-cohort) was significantly better than the age-period (AP) and age-cohort (AC) models. The deviance information criterion (DIC) of the APC model was 38.79, indicating a good fit of the model compared with other submodels (143.40 for AC, 139.18 for AP). Accordingly, our subsequent estimations were based on the APC models.

**Table 2:** Comparison of age-period-cohort submodels for lung cancer incidence

Terms in model	Resid. DF	Residual	Residual Deviance	Deviance	***P*** value
Age	173	1074.14			
Age-drift	172	728.96	1	345.18	*P* < 0.001
Age-Cohort	167	585.56	5	143.40	*P* < 0.001
Age-Period-Cohort	162	546.77	5	38.79	*P* < 0.001
Age-Period	167	685.96	5	139.18	*P* < 0.001
Age-drift	172	728.96	5	43.01	*P* < 0.001

Resid. DF: residual degrees of freedom

**Fig. 1:**
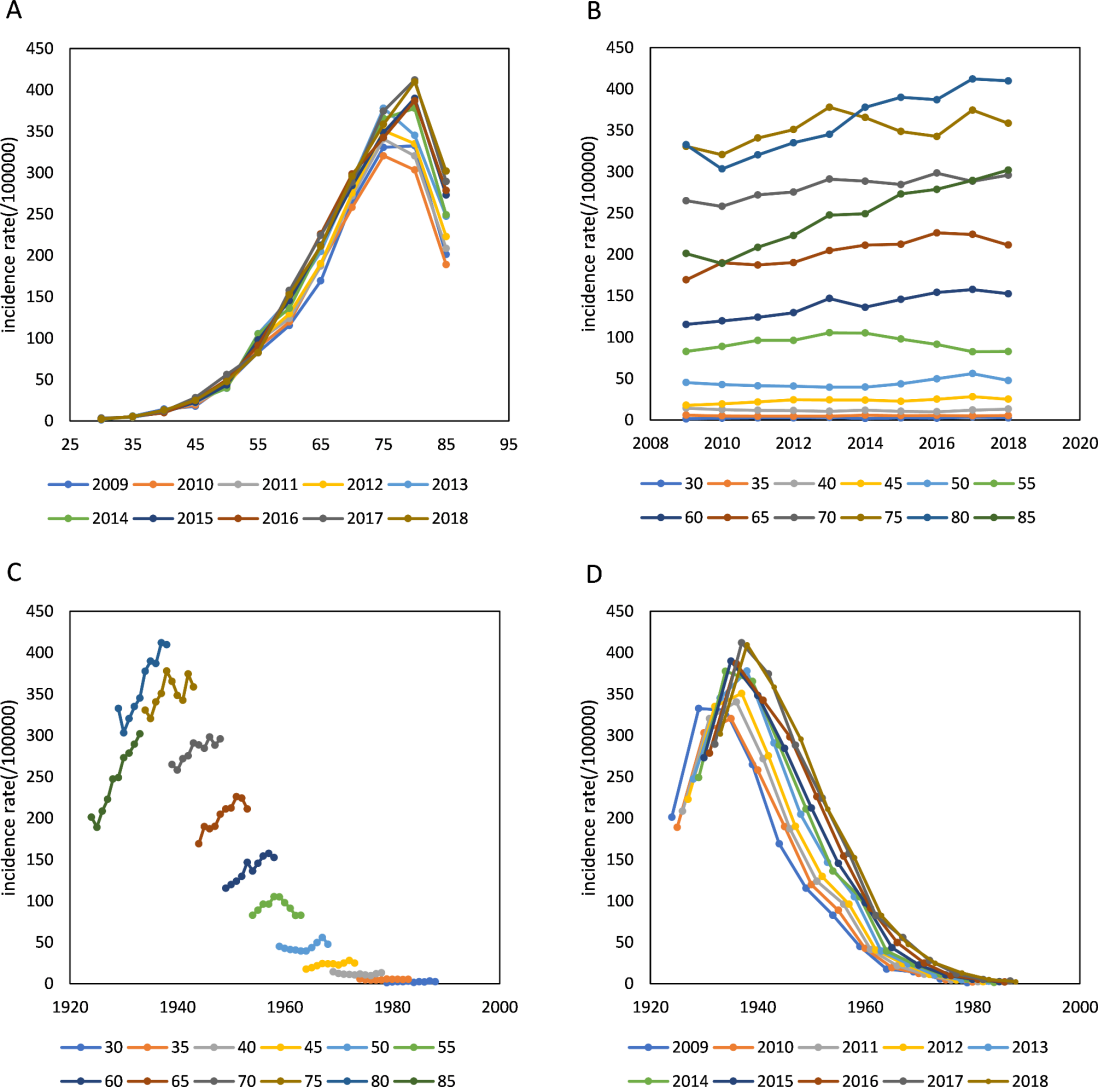
Incidence of lung cancer per 100,000 by age, period and cohort effect (A: age trend by period; B: period trend by age; C: cohort trend by age; D: cohort trend by period)

Figure 1 shows the ASR of lung cancer according to age, period, and cohort effects. All observed rates were reported in 1-year periods and 5-year age groups (30 to 85 every 5 years, and 85 years and older; the age group under 30 years of age was excluded due to sparse cases). The incidence rates increased with age until 80 years in every period (Fig. 1 A). While the incidence rate remained low in the age group under 55 years and showed a stable trend with the period, it increased with the period among age groups above 55 years. The greatest increase was among those aged 80 years and older. (Fig. 1B). Cohort trends suggested that the cohort effects increased across age groups but decreased sharply within each period (Fig. 1 C, 1D).

## Predicted lung cancer incidence in Jiangsu, 2019–2030

**Fig. 2:**
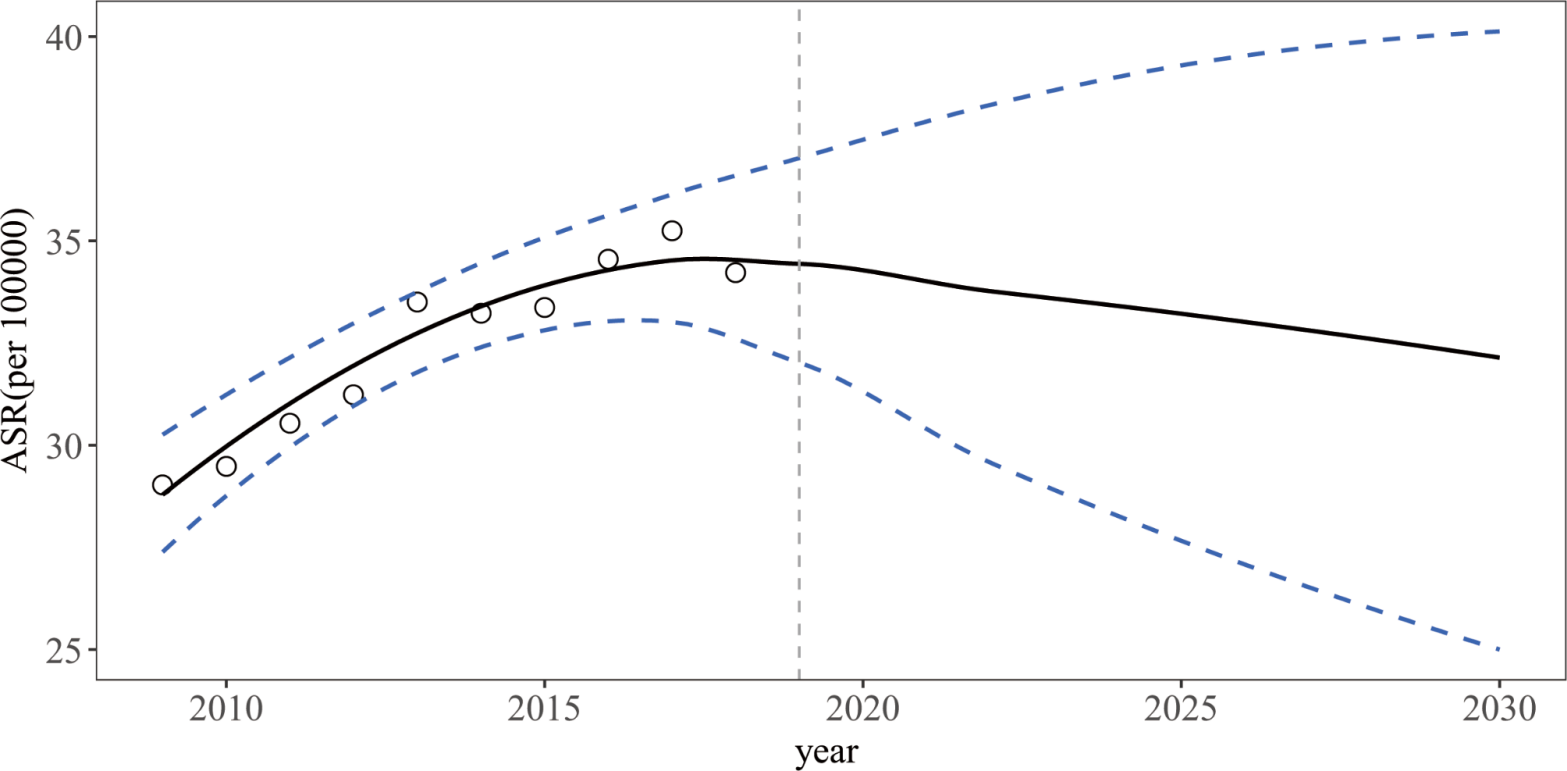
Observed and predicted lung cancer incidence from 2009–2030 in Jiangsu (The open dots represent the observational values, and the blue dashed line denotes the 95% highest density interval of prediction values. The predictive mean value is shown as a black solid line. The vertical dashed line indicates where the prediction starts.)

**Fig. 3:**
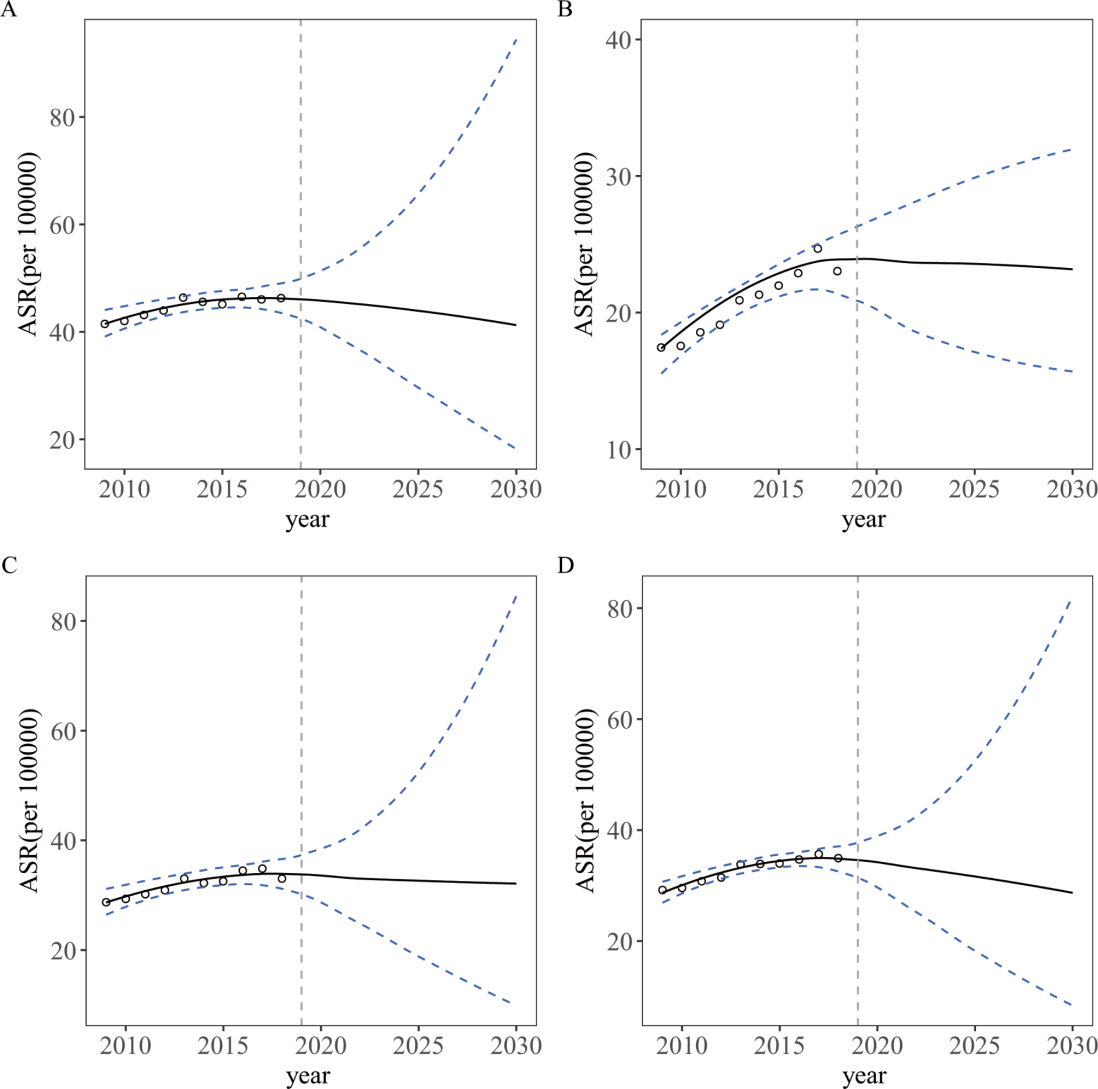
Observed and predicted lung cancer incidence from 2009–2030 in both sexes and urban and rural areas of Jiangsu (A: males; B: females; C: urban; D: rural)

Considering the different distributions of lung cancer with respect to sex and region, we predicted the ASR stratified by sex and region with the use of the BAPC model. The predicted incidence rates from 2019 to 2030 are shown in Table 3 separately and by male and female populations and urban and rural regions. The ASR of lung cancer will decrease slightly to 32.14 per 100,000 (95% HDI, 24.99, 40.22) during this period (AAPC=-0.55%, 95% CI, -0.69%, -0.41%) (Table 3; Fig. 2). A downward trend is expected for both sexes, but it will be more pronounced in males (AAPC=-0.89%, 95% CI, -1.11%, -0.68%) than in females (AAPC=-0.02%, 95% CI, -0.28%, 0.24%) (Table 3; Fig. 3 A, 3B). Meanwhile, the incidence of lung cancer is expected to decrease in both urban and rural regions, but this trend is more pronounced in rural regions (AAPC=-1.72%, 95% CI, -2.17%, -1.26%) than in urban regions. (AAPC=-0.42%, 95% CI, -0.55%, -0.30%) (Table 3; Fig. 3 C, 3D).

**Table 3:** Age-standardized incidence of lung cancer from 2019 to 2030 in Jiangsu

	2019 [per 100,000 (95% HDI)]	2030 [per 100,000 (95% HDI)]	2019–2030 AAPC [% (95% CI)]
Overall	34.08 (31.50, 36.85)	32.14 (24.99, 40.22)	-0.55 (-0.69, -0.41) ^*^
Sex			
male	45.81 (42.47, 49.42)	41.28 (18.47, 95.08)	-0.89 (-1.11, -0.68) ^*^
female	23.14 (20.46, 26.04)	23.12 (15.57, 32.01)	-0.02 (-0.28, 0.24) ^*^
Region			
urban	33.40 (30.06, 36.90)	32.18 (10.25, 85.81)	-0.42 (-0.55, -0.30) ^*^
rural	34.36 (31.47, 37.31)	28.48 (8.73, 82.11)	-1.72 (-2.17, -1.26) ^*^

^*^P < 0.001

## Discussion

In the current study, we analysed the temporal trends and a prediction of future trends in lung cancer incidence rates for the next decades in Jiangsu. Several messages can be derived from our study. By applying BAPC models, we found that the trends of incidence were mainly driven by ageing effects. The incidence rates of lung cancer have increased in the past decade; in contrast, they will show a downward trend in the next 12 years (2019–2030). The future downward trend will be consistent in males and in rural regions, while incidence rates will be stable in females and in urban regions.

The incidence of lung cancer was higher in males than in females in Jiangsu. This is consistent with the results of previous studies at the national and provincial levels [26, 27]. The reasons for the higher incidence in males are probably due to the different smoking rates between males and females: 44.0% of males and 1.6% of females smoked in Jiangsu in 2017 [5]. The duration of smoking should be considered the strongest determinant of lung cancer risk in smokers [28]. Previous studies have shown that occupational exposure is also a major risk factor for lung cancer [29, 30]. Due to their occupational choices, males are exposed to jobs with greater exposure to risk factors [31]. This may also lead to a higher incidence of lung cancer in males. However, the upward trend of lung cancer incidence was more pronounced in females than in males. Passive smoking and cooking smoke in the indoor environment may be responsible for the increased incidence in females [26, 32]. Furthermore, females have a higher risk of developing lung cancer than males when they are also smokers [33].

The incidence of lung cancer was higher in more developed regions than in less developed regions worldwide [1, 34]. However, our study showed that there was a higher incidence of lung cancer in rural regions than in urban regions. Moreover, the increase in lung cancer incidence in rural regions was also higher than that in urban regions. This may be related to the rising prevalence of smoking among rural men and an increase in their daily cigarette consumption [35]. In addition, the higher incidence in rural areas may be related to the differences in environment and lifestyle, such as urban-rural differences in solid fuel use and domestic water use [36, 37]. At the same time, atmospheric monitoring networks and management tools are becoming increasingly sophisticated in urban areas, while rural areas are relatively poorly equipped [38].

With BAPC models, we were able to identify the effects of age, period, and cohort on the outcome of cancer incidence, which is the first step in exploring the causal processes of the disease [25]. Age is often the main factor in BAPC analysis, as it accounts for consistent extrinsic factors, such as the accumulative exposure to factors [39]. The period effect accounts for all factors that affect every person during a time period in history, such as pollution or medical interventions [40]. The cohort effect accounts for events that affect generations, such as malnutrition of children during wars or changing habits [12, 41]. Our results show that the age effect on lung cancer incidence was seen mainly in the elderly population, which might be related to China’s ageing population [42]. In addition, the age effect is likely explained by age-related causes, such as accumulative exposures of the body to carcinogens over time and the accretion of mutations [43, 44]. The period effect may be related to the more severe air pollution and high smoking rate in China in recent years [45, 46]. Meanwhile, the cohort effect may be related to a higher level of education and awareness of disease prevention and control in the newer birth cohort [47].

The BAPC model provided very reliable and stable estimations for disease prediction [48–50]. Based on the assumption that past age, period, and cohort trends would continue, we made lung cancer projections for the next 12 years in Jiangsu Province. Our results show that the age-standardized incidence of lung cancer in Jiangsu will continue to decrease until 2030. This trend was maintained in men and rural areas, while it was not seen in women and urban areas. Although the disease burden of lung cancer in Jiangsu Province is lower than at the national level [51], it shows similar trends both at the national level and at the provincial level [7, 8, 52, 53]. This may be related to the more pronounced ageing population trends in women and more developed regions [54, 55], in addition to air pollution in urban areas, which also contributes to the high incidence of lung cancer [45, 56]. Additionally, in recent years, the biological basis of higher genetic susceptibility and oestrogen exposure in females has attracted the attention of researchers [57, 58].

The main risk factors for lung cancer include smoking, atmospheric pollution and occupational exposure [59, 60]. As China is currently the largest producer and consumer of tobacco, the government should introduce policies to limit and reduce smoking rates, such as increasing taxes on tobacco and banning smoking in public places [61]. Air pollution is also an issue that deserves attention, as air pollutants are now classified as major carcinogens [62]. According to the Report on the State of the Ecology and Environment in China 2020, 40.1% of cities in China failed to meet national air quality standards [38]. This requires the government to strengthen atmospheric control, encourage low-carbon travel and reduce carbon emissions. For occupational exposures, regular health check-ups, such as low-dose computed tomography scans, can provide better prevention of lung cancer.

To the best of our knowledge, we were able to predict the lung cancer incidence up to 2030, providing evidence for future policy-making. Several limitations should be noted before interpreting our results. First, underreporting and failure of diagnosis can occur in cancer registration, particularly in less developed areas, and therefore some of these lung cancer estimates may suffer from underestimation [63]. Secondly, these estimates of future incidence should not be overinterpreted, as they were based on the incidence data from only a short time period.

## Conclusion

In summary, a consistent decrease is expected in the incidence of lung cancer during the next decades. The decrease in incidence will be more pronounced in males and rural regions but will be stable in females and urban regions. The results will be helpful in understanding the current situation of lung cancer in Jiangsu in depth and will provide essential information for health-related staff to monitor and control the disease.

## Data Availability

The datasets used and analysed during the current study are available from the corresponding author on reasonable request.

## Abbreviations

AAPC: average annual percentage change
ASR: age-standardized rate
CI: confidence interval
HDI: highest density interval
BAPC: Bayesian age-period-cohort
